# ParB Partition Proteins: Complex Formation and Spreading at Bacterial and Plasmid Centromeres

**DOI:** 10.3389/fmolb.2016.00044

**Published:** 2016-08-29

**Authors:** Barbara E. Funnell

**Affiliations:** Department of Molecular Genetics, University of TorontoToronto, ON, Canada

**Keywords:** chromosome dynamics, segregation, ParABS, DNA-binding, bridging

## Abstract

In bacteria, active partition systems contribute to the faithful segregation of both chromosomes and low-copy-number plasmids. Each system depends on a site-specific DNA binding protein to recognize and assemble a partition complex at a centromere-like site, commonly called *parS*. Many plasmid, and all chromosomal centromere-binding proteins are dimeric helix-turn-helix DNA binding proteins, which are commonly named ParB. Although the overall sequence conservation among ParBs is not high, the proteins share similar domain and functional organization, and they assemble into similar higher-order complexes. *In vivo*, ParBs “spread,” that is, DNA binding extends away from the *parS* site into the surrounding non-specific DNA, a feature that reflects higher-order complex assembly. ParBs bridge and pair DNA at *parS* and non-specific DNA sites. ParB dimers interact with each other via flexible conformations of an N-terminal region. This review will focus on the properties of the HTH centromere-binding protein, in light of recent experimental evidence and models that are adding to our understanding of how these proteins assemble into large and dynamic partition complexes at and around their specific DNA sites.

In bacteria, the segregation, or partition, of low-copy-number plasmids and cellular chromosomes depends on the activity of site-specific DNA binding proteins to recognize one or more copies of a centromere-like DNA site. These “centromere-binding proteins” generally work in concert with an ATPase or GTPase, resulting in dynamic movement and positioning of plasmids or chromosomal domains during the cell cycle (reviewed in Wang et al., 2013; Baxter and Funnell, 2014; Bouet et al., 2014). Centromere-binding proteins fall into one of two structural classes, as helix-turn-helix (HTH), or ribbon-helix-helix site-specific DNA binding proteins. In bacteria, the proteins of all chromosomal, and many plasmid partition systems are members of the HTH class. They share similar properties *in vivo* and *in vitro*, including similar domain organization and DNA-binding properties, although there are also interesting differences. They all form large partition complexes *in vivo* that can be visualized as foci using fluorescence approaches. This review will focus on the properties of the HTH centromere-binding proteins, and in particular, how they assemble into large partition complexes. I will discuss the contribution of protein domains, the “spreading” phenomenon that has been reported as a general property of HTH centromere-binding proteins, and how flexibility in the protein allows multiple conformations and binding modes in complex assembly.

The components of partition systems are commonly named ParA (the partition ATPase), ParB (the centromere-binding protein), and *parS* (the centromere or partition site). Plasmids typically contain one *parS* located near the *parA* and *parB* genes, although some have multiple *parS* sites. Bacterial chromosomes contain several *parS* sites, which are primarily located in the chromosomal domain that contains the replication origin. Bacterial ParA and ParB are also called Soj and Spo0J, respectively, because their genes were first defined by roles in sporulation of *Bacillus subtilis* (Ireton et al., 1994). For simplicity, I will use the ParABS nomenclature, with specific names for some of the discussion to be consistent with published literature.

HTH ParBs share a similar domain organization although the primary sequence conservation is not high among members of this family. In general, the protein is divided into three regions: A central HTH DNA binding domain is flanked by a C-terminal dimer domain and an N-terminal region necessary for protein oligomerization (Figure 1A). Flexible linkers connect the domains, and flexibility in domain organization, orientation, and folding have been observed in biochemical experiments and crystal structures. The most highly conserved sequence among plasmid and chromosomal ParBs is a short arginine-rich motif in the N-terminus, which is often called an arginine patch (Yamaichi and Niki, 2000). ParA interactions are often specified by residues near the N-terminus of ParB (Radnedge et al., 1998; Figge et al., 2003; Leonard et al., 2005; Ah-Seng et al., 2009), although there are exceptions. For example, the ParA interactions for RK2 KorB and *Pseudomonas aeruginosa* ParB map to the center and dimer domain, respectively (Lukaszewicz et al., 2002; Bartosik et al., 2004). There are also added complexities to the general arrangement. For example, P1 ParB and its relatives contain an additional site-specific DNA binding activity within the dimer domain. These ParBs recognize both an inverted repeat and a second DNA motif in their *parS* sites, via their HTH domain and dimer domains, respectively (Schumacher and Funnell, 2005).

**Figure 1:**
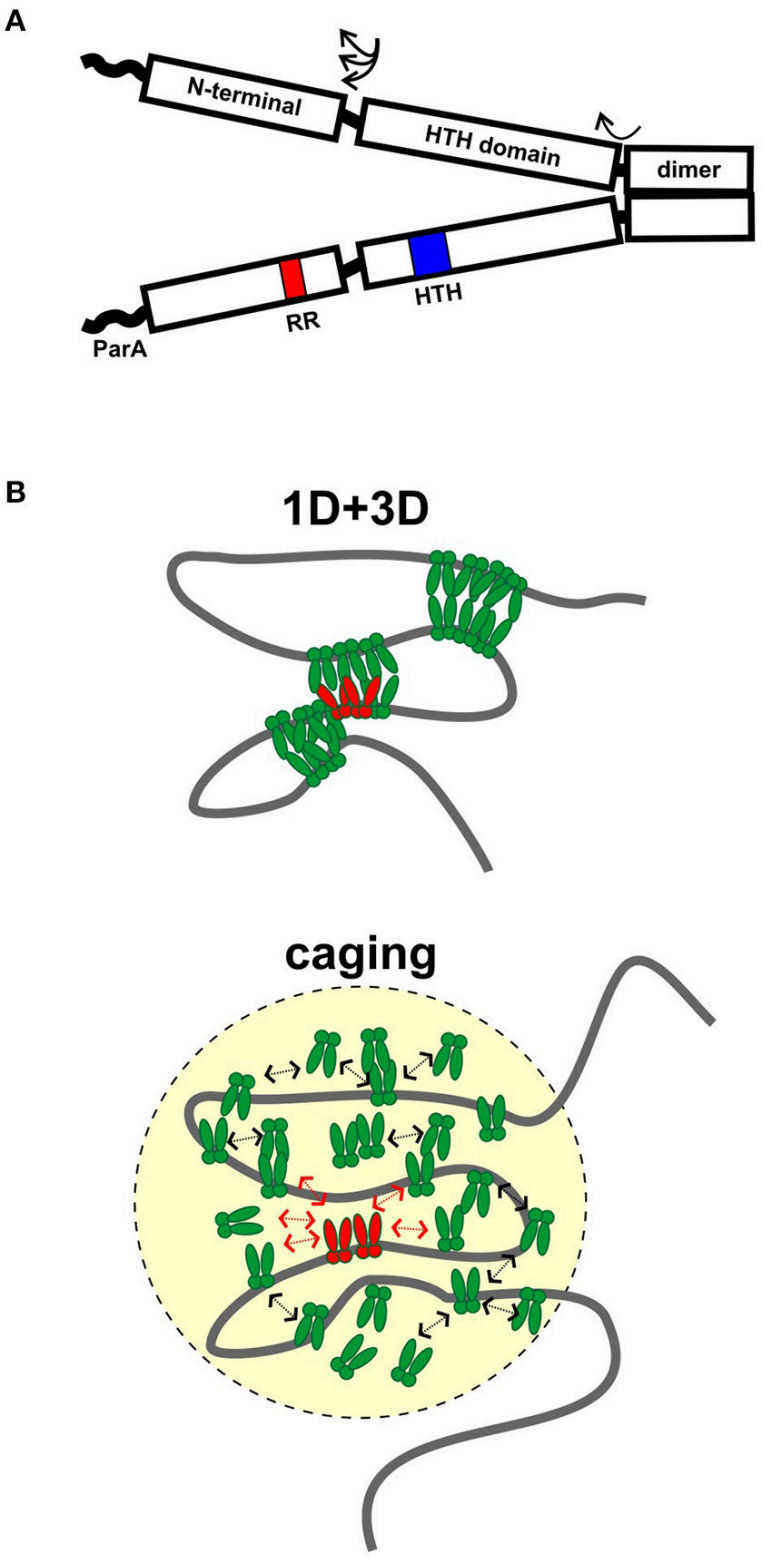
**Assembly of ParB partition complexes. (A)** Cartoon of the conserved domain structure of HTH ParBs, shown as a dimer. The black rectangles represent the regions of the protein for which there is some structural information: the C-terminal dimer domain, the HTH DNA binding domain, and the N-terminal domain. The three regions are connected by flexible linker sequences (arrows). The linker length here is represented as short, as in the *Hp*Spo0J and P1 ParB published structures (Schumacher and Funnell, 2005; Chen et al., 2015), but may be longer in other ParBs. The wavy line represents the region that interacts with ParA in many, although not all, ParBs. The position of the HTH motif (blue) and the conserved arginine patch motif (RR, red) are indicated in one monomer. **(B)** Diagrams of 1D + 3D and caging models for higher-order ParB binding and partition complex assembly. ParB dimers bound to *parS* (in red) nucleate complex assembly and interact with other ParBs in green. Arrows in the caging architecture illustrate that dynamic associations maintain the cluster of ParB.

## Partition complex assembly, spreading, and bridging

Partition complex assembly begins with the recognition of *parS* by a dimer of ParB, followed by loading of multiple ParB dimers to form a very large protein-DNA complex (Baxter and Funnell, 2014). These higher-order complexes are necessary as both the substrates and the activators of the mechanisms of partition. The number of ParB protein foci observed inside cells is usually lower than the number of *parS* sites, leading to the idea that inter and intra-molecular pairing of *parS* sites occurs in plasmids and chromosomal domains.

Spreading is an unusual feature for site-specific DNA binding proteins that is common to HTH ParBs, and it reflects how ParB assembles into higher-order complexes (Rodionov et al., 1999; Murray et al., 2006; Breier and Grossman, 2007; Sanchez et al., 2015). Measured by ChIP approaches, *in vivo* ParB binding extends beyond *parS* into the surrounding non-specific DNA, often many kb away from the site. Binding is maximal at *parS*, and diminishes non-linearly as a function of distance from *parS*. Spreading can be impeded by “roadblocks,” which are strong binding sites for other proteins (Rodionov et al., 1999; Murray et al., 2006). Spreading has also been inferred from the ability of some ParBs, especially when overexpressed, to silence expression of nearby genes (Lynch and Wang, 1995; Rodionov et al., 1999; Bartosik et al., 2004; Bingle et al., 2005; Kusiak et al., 2011). Silencing is likely a consequence of protein overexpression and is not necessary for partition (Rodionov and Yarmolinsky, 2004). Spreading ability is required however, as ParB mutants that do not spread are defective in partition (Rodionov et al., 1999; Autret et al., 2001; Breier and Grossman, 2007; Kusiak et al., 2011; Graham et al., 2014). In particular, the arginine patch in ParB is essential for spreading, focus formation, and partition activity *in vivo*.

The first and simplest model described spreading as lateral protein-protein association along the DNA as a one-dimensional filament (Rodionov et al., 1999). However some properties of ParBs lead investigators to question this idea. Studies with plasmid KorB and with *B. subtilis* Spo0J (*Bs*Spo0J) argued that the intracellular concentration of ParB was insufficient to account for the amount of spreading observed *in vivo* if arranged as a one-dimensional filament (Bingle et al., 2005; Graham et al., 2014). It was also difficult to demonstrate biochemically that ParB binding to *parS* increased the affinity of ParB for adjacent non-specific DNA.

Two other models have emerged recently, based on sophisticated microscope and ChIP-seq technologies as well as computer modeling and traditional biochemistry (Broedersz et al., 2014; Graham et al., 2014; Sanchez et al., 2015). The first proposes that limited lateral ParB-ParB interactions (1D) in combination with inter and intra-molecular looping and bridging (3D) act to build large complexes and coalesce many ParB molecules into foci (Broedersz et al., 2014; Graham et al., 2014; Figure 1B). Computer modeling was used to argue that neither 1D nor 3D interactions alone could generate ParB foci; that the 1D + 3D arrangement allows focus formation because it creates a surface tension on the ParB cluster that counteracts the tendency for entropy to disperse the protein on DNA (Broedersz et al., 2014). Elegant *in vitro* TIRF microscopy experiments provided experimental support for the bridging activity: flow-stretched DNA was condensed by *Bs*Spo0J in a manner that is most consistent with ParB bridging across loops within the same or across different DNA molecules (Graham et al., 2014). The experiments were however unable to demonstrate any sequence-specificity for *parS*, leading to the suggestion that experiments on flow-stretched DNA were not recapitulating an undefined aspect of critical nucleation properties of ParB bound to *parS*. For example, one factor missing from these experiments is DNA supercoiling, which affects chromosome compaction and may strongly influence the DNA binding properties of ParB in higher-order complexes.

Mutations in conserved arginine residues of the arginine patch motif eliminated bridging in the TIRF assay, consistent with the requirement for this motif for spreading and partition *in vivo* (Graham et al., 2014). Interestingly, *Bs*Spo0J mutated at one residue in the arginine patch (G77S), which is unable to form foci or spread *in vivo*, was still able to bridge DNA, and with slightly higher stability than that of wild-type *Bs*Spo0J. Therefore the bridging activity of ParB is necessary but not sufficient for complex formation. These observations lead to the suggestion that the G77S mutation may promote inappropriate bridging and/or alter the dynamics of bridging necessary for proper complex assembly *in vivo*. Modeling the spreading/bridging behavior also predicted that roadblocks would decrease the probability of loops forming in their vicinity, interfering with complex assembly beyond the roadblock (Broedersz et al., 2014).

In contrast, a second model proposes that a network of stochastic binding of ParB explains the clustering of ParB molecules around *parS* (Sanchez et al., 2015). In essence, the nucleation of ParB by *parS* creates, and maintains a very high localized concentration of ParB in a “cage” by many weak but dynamic interactions with itself (dimer-dimer interactions via the N-terminal domains), as well as with non-specific DNA around *parS* (Figure 1B). In caging, these interactions do not need to occur simultaneously or to bridge DNA (Figure 1B). Computer modeling of the patterns of ParB occupancy around *parS* measured by ChIP was used to argue that they are not consistent with either 1D lateral spreading or a combination of 1D spreading and 3D bridging. Biochemical examination showed no evidence that binding of one ParB could stabilize binding of an adjacent ParB. The caging model neither requires nor excludes bridging, although bridging interactions are intuitively attractive as part of the dynamic glue. The model does depend on other properties of the DNA chromosome, such as topology or organization by other nucleoid-binding proteins *in vivo* to help restrict the DNA within the cage. Roadblocks could alter local DNA organization and reduce the proximity of *parS* to the rest of the DNA in three-dimensional space; that is, place this DNA outside the cage.

Both models agree that ParB binding to *parS* must nucleate the formation of higher-order complexes to explain ParB clustering and foci *in vivo* (Broedersz et al., 2014; Sanchez et al., 2015). This is most simple to envision in plasmid systems such as P1, in which ParB's affinity for *parS* is at least 10,000-fold higher than that for non-specific DNA (Funnell, 1991). However this affinity difference is small for some ParBs (Broedersz et al., 2014; Taylor et al., 2015), and it was suggested that a conformational change is induced in ParB by *parS*-specific binding to effectively anchor the focus at the site. Both models agree that multiple ParB-ParB interaction interfaces must be involved in assembly of higher-order partition complexes, and that the dynamics of these interactions are critical for proper assembly and function in partition. How bridging activities detected *in vitro* contribute to ParB activity *in vivo* remains to be resolved. Further refinement of these models will depend on the ability to completely reconstitute the *parS*-dependent complex assembly *in vitro*, which will in turn depend on identifying the other factors necessary for caging or bridging, and on the nature of ParB-ParB and ParB-DNA interactions at the molecular level. The influence of ParA on complex architecture and dynamics has also yet to be defined (see below).

## ParB-ParB and ParB-DNA interactions in higher-order-complex assembly

Site-specific DNA binding of ParBs to cognate *parS* sites has been examined directly and in detail in many different partition systems, but the *parS*-dependent formation of higher order, large partition complexes has been difficult to reconstitute *in vitro.* However, there are insights arising from structural biology of several plasmid and chromosomal ParBs, which are leading to a preliminary, albeit incomplete, picture of partition complex assembly.

Although there are no structures of a full length ParB, those of individual domains or combinations of domains, with and without *parS* DNA, have provided clues concerning the three dimensional organization of the protein with respect to DNA and to itself. There are structures of the HTH domains of three plasmid ParBs (P1 ParB, F SopB, and RP4 KorB) in complex with their specific DNA sites, and of their dimer domains (SopB and KorB dimer domains solved separately from the HTH; Delbrück et al., 2002; Khare et al., 2004; Schumacher and Funnell, 2005; Schumacher et al., 2010). Structures of two chromosomal ParB fragments, each containing the N-terminal region and adjacent HTH domain, have visualized the oligomerization interactions of the proteins. These ParBs are *Thermus thermophilus* Spo0J (*Tt*Spo0J) and *Helicobacter pylori* Spo0J (*Hp*Spo0J); structure of the latter was solved bound to *parS* (Leonard et al., 2004; Chen et al., 2015).

One of the first themes to highlight is that of flexibility. Taken together, the structures indicate that these three regions of the protein are connected by flexible linkers, and that their orientation with respect to each other can vary. The conformation of the N-terminus is particularly flexible (Chen et al., 2015).

**Figure 2:**
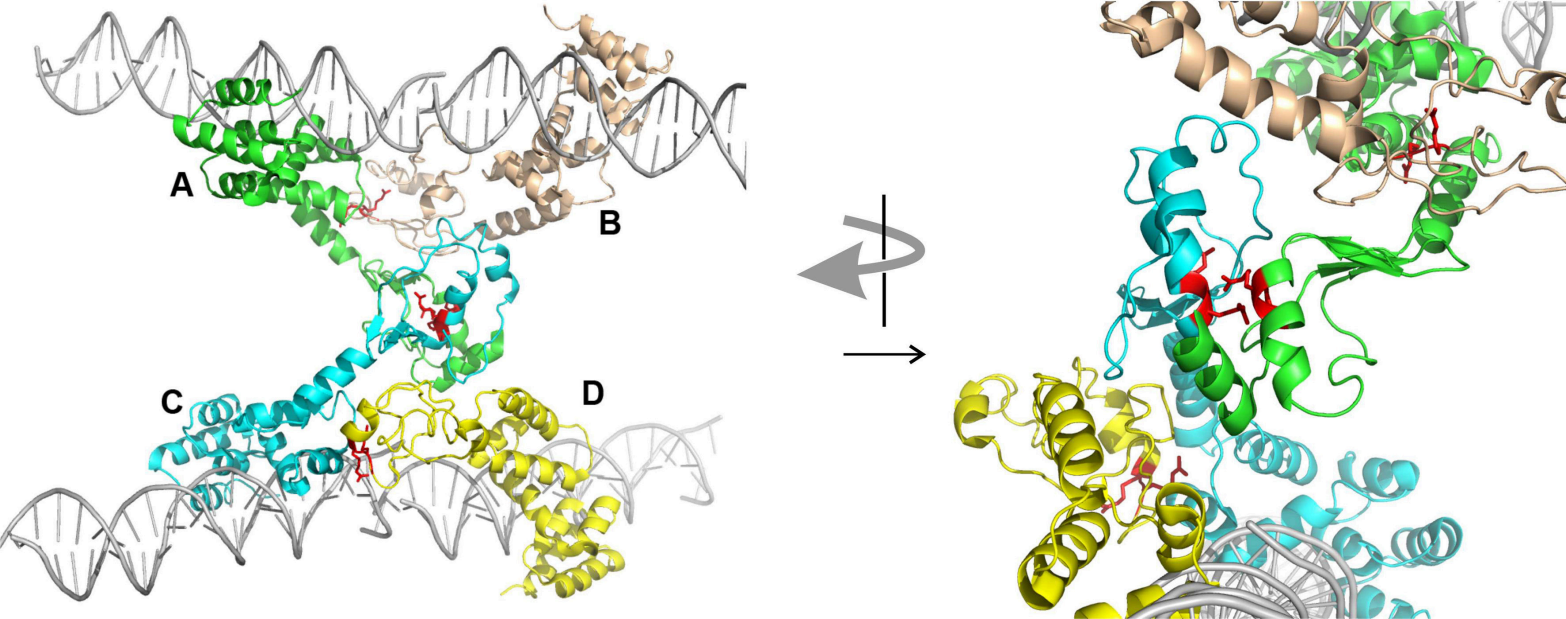
**Structure of *Hp*Spo0J monomers (lacking C-terminal dimer domain) bound to and across *parS* DNA (PDB 4UMK, Chen et al., 2015)**. Four monomers (A to D) make adjacent (AB and CD) and transverse (AC) interactions. The arginine patch motif is illustrated by two red arginines from each monomer. The arrangement on the left is rotated approximately 90° and magnified on the right to illustrate the environments of these arginines in the different interactions. The images were generated using the PyMOL Molecular Graphics System, Version 1.8.2.1 Schrödinger, LLC.

Second, ParBs are bona-fide HTH site-specific DNA binding proteins, but with a twist. As expected, the HTH domains contact inverted repeat sequences within *parS* via helix insertion into the major groove of DNA. However, unexpected features emerged from the structures. First, residues outside of the recognition helix also contribute to specificity for *parS* in SopB, KorB, and *Hp*Spo0J (Khare et al., 2004; Schumacher et al., 2010; Sanchez et al., 2013; Chen et al., 2015). Second, both the P1 ParB and *Hp*Spo0J structures demonstrated bridging across *parS* sites mediated by the dimer and N-terminal domains, respectively (Schumacher and Funnell, 2005; Chen et al., 2015). Each monomer of a P1 ParB dimer interacts with a half-site on a different DNA molecule, effectively pairing two *parS* sites. *Hp*Spo0J-*parS* bridging interactions are mediated by the N-terminal oligomerization regions of the protein (Figure 2). Four monomers of *Hp*Spo0J (monomeric because it lacks the C-terminal dimer domain) interact with two *parS* oligos and with each other in a cross-bridge arrangement across the DNA molecules (molecules A–D in Figure 2). The monomers share a common HTH domain, but show different conformations in the extended N-terminal regions as well as different interactions with each other. There are two adjacent (AB and CD) and one transverse (AC) sets of protein-protein interactions, which are distinct. For example, the conserved arginine patch motifs are close to each other at the AC interface, but not at the AB or CD interfaces (Figure 2). In the structure, there is no BD interaction, leaving these surfaces available, perhaps for interactions with different conformations of ParB or with different partners.

The overall fold of *Hp*Spo0J is similar to that of *Tt*Spo0J, except for a bend in the linker between the N-terminal and HTH domains (Leonard et al., 2004; Chen et al., 2015). It was suggested that the *Tt*Spo0J structure may represent a closed conformation that opens up following DNA binding to the *Hp*Spo0J architecture.

How do these structures inform us of higher-order complex assembly, particularly when ParB binds, bridges, and spreads on non-specific DNA adjacent to and away from *parS*? The simplest model is that the HTH is responsible for both specific and non-specific DNA interactions, which is supported by the observation that a triple substitution in the HTH domain of F SopB impairs both DNA binding activities (Ah-Seng et al., 2009). In this case the *Hp*Spo0J-DNA structure may represent ParB-ParB and ParB-DNA interactions during spreading at any DNA site. The requirement for the N-terminus in higher-order complex formation *in vivo* is also consistent with this picture. The flexibility of and variation in the cross-monomer interactions and interfaces seen in the *Hp*Spo0J structure make it an attractive model for the ability of ParB to make multiple and flexible interactions during complex assembly and maintenance. However, one recent study suggests that the specific and non-specific DNA binding activities may be distinct (Taylor et al., 2015). The specific and non-specific DNA binding activities of *Bs*Spo0J showed different properties *in vitro*, including different abilities to protect the HTH domain from proteolysis. The results lead to the proposal that the N-terminus contains a DNA binding region that is distinct from the HTH. These observations may also reflect differences between plasmid and chromosomal ParBs. We must await identification of the protein-DNA contacts at non-specific sites before we can confirm the organization of ParB during higher-order partition complex assembly.

## The role of ParA in ParB-DNA complexes

During partition, ParAs form patterns on the surface of the bacterial nucleoid due to dynamic interactions with ParB bound to *parS* (Hatano et al., 2007; Ringgaard et al., 2009; Hatano and Niki, 2010; Ah-Seng et al., 2013). The patterning is necessary for the segregation of plasmids and chromosomal domains, and the molecular mechanisms involved are still being defined. ParA is not necessary to form the large ParB-DNA complexes seen *in vivo* and *in vitro*, but ParA can influence or modulate these complexes. For example, the behavior of several *parA* and *parB* mutants supports a proposal that ParA is necessary to separate pairs or groups of plasmids during segregation (Fung et al., 2001; Ah-Seng et al., 2013). For the ParBs that interact with ParA via N-terminal regions that are adjacent to the flexible ParB-ParB oligomerization interface, one attractive idea is that ParA-ParB interactions at the N-terminus may influence the available conformations for ParB-ParB interactions next door. For example, specific interference with the transverse ParB-ParB interactions in the *Hp*Spo0J structure might favor intramolecular associations over intermolecular ones (Figure 2).

## Flexibility and order in partition complex assembly

Taken together, the biochemistry and structural biology of the N-terminal regions of ParBs imply that the folding and structures are flexible, dynamic, and fluid. Recent experiments using magnetic tweezers support the idea that the complexes are not highly ordered (Taylor et al., 2015). ParB-ParB (dimer-dimer) interactions via the N-terminal domain must contribute to higher-order complex assembly and function. It is attractive to consider that the flexibility of the N-terminus, in folding and conformation, is important for the dynamics and architecture of the large higher-order partition complex *in vivo*. This conformational flexibility resembles the properties of so-called “intrinsically disordered” proteins and domains whose unstructured properties allow proteins to sample and bind to multiple targets and in multiple ways (Wright and Dyson, 1999; Uversky, 2016). The same region in ParB could be involved, directly or indirectly, in different binding scenarios with different partners, including ParA, and potentially non-specific DNA. Why are flexibility, dynamics, and size important for these partition complexes? ParBs are engaging in multiple and constantly changing interactions during partition. Clustering creates a condensed, organized DNA substrate and provides a high density of ParBs available to ParA. Defining the molecular nature of these interactions continues to be an essential step toward the understanding of these intriguing DNA binding proteins.

## Author contributions

The author confirms being the sole contributor of this work and approved it for publication.

## Funding

This work was supported by Canadian Institutes of Health Research grant 133613.

### Conflict of interest statement

The author declares that the research was conducted in the absence of any commercial or financial relationships that could be construed as a potential conflict of interest.
